# Peptidyl Activity-Based Probes for Imaging Serine Proteases

**DOI:** 10.3389/fchem.2021.639410

**Published:** 2021-04-29

**Authors:** Paulina Kasperkiewicz

**Affiliations:** Department of Chemical Biology and Bioimaging, Wroclaw University of Science and Technology, Wroclaw, Poland

**Keywords:** activity-based probes, serine proteases, imaging, chemical reagents, internally quenched fluorogenic substrates, enzyme detection

## Abstract

Proteases catalyze the hydrolysis of peptide bonds. Products of this breakdown mediate signaling in an enormous number of biological processes. Serine proteases constitute the most numerous group of proteases, accounting for 40%, and they are prevalent in many physiological functions, both normal and disease-related functions, making them one of the most important enzymes in humans. The activity of proteases is controlled at the expression level by posttranslational modifications and/or endogenous inhibitors. The study of serine proteases requires specific reagents not only for detecting their activity but also for their imaging. Such tools include inhibitors or substrate-related chemical molecules that allow the detection of proteolysis and visual observation of active enzymes, thus facilitating the characterization of the activity of proteases in the complex proteome. Peptidyl activity-based probes (ABPs) have been extensively studied recently, and this review describes the basic principles in the design of peptide-based imaging agents for serine proteases, provides examples of activity-based probe applications and critically discusses their strengths, weaknesses, challenges and limitations.

## Introduction

Proteases comprise approximately 700 enzymes and constitute ∼2% of the human genome (Rawlings et al., 2002). In humans, these enzymes are divided into five classes according to their catalytic mechanism: aspartyl, cysteine, metallo-, serine, and threonine proteases. These five classes are further divided into smaller groups called families (Rawlings et al., 2002), among which cysteine and serine proteases are the most numerous.

The peptide processing by cysteine, serine and threonine proteases utilizes the charge relay system that supports the breakdown of the substrate sequence (Hedstrom, 2002). The newly formed peptides are mediators in catalytic cascades such as the blood coagulation system, where the cascade of the activation of serine proteases triggers a series of events leading to blood coagulation. Other examples include the role of neutrophil serine proteases (NSPs) in the immune response (Nauseef and Borregaard, 2014; Pham, 2006) and granzymes (Grs) in programmed cell death (Trapani et al., 1998; Trapani and Smyth, 2002; Trapani and Sutton, 2003; Susanto et al., 2012). Substrate hydrolysis by serine proteases is irreversible; therefore, their physiological activity is tightly regulated at three stages. The first level is the expression, the second is activation by posttranslational processing and the third is inactivation by endogenous inhibitors of serine proteases, called serpins (Silverman et al., 2001). The majority of serine proteases are synthesized as inactive zymogenes that require posttranslational modification(s) for activation (Hedstrom, 2002). Processing of the zymogen is mainly performed by other proteases that remove N- and/or C-terminal signaling peptides in the protease structure. If all of the control points fail, the uncontrolled activity of serine proteases led to the undiserable hydrolysis of peptides that is associated with diseases, such as chronic obstructive pulmonary disease (COPD), cancer progression, and metastasis (Jenne and Kubes, 2016; Pandey et al., 2017).

Proteases are important in both health and disease, and understanding their biological functions is indisputably significant for diagnosis and personal treatment. Therefore, as active serine proteases play both positive and negative roles, targeting their activity is required rather than monitoring their expression level. Since serine proteases compose one-third of all proteases and have been found to be key players in many diseases (López-Otín and Bond, 2008; Heutinck et al., 2010; Korkmaz et al., 2010; McKelvey et al., 2020), active serine proteases serve as a new category of biomarkers for personalized treatment that need to be evaluated. To establish the accurate function of serine proteases, new methods focused on investigating enzyme activity are required. As the cellular localization of active enzymes is fundamental, antibody-related techniques are now supplemented with activity-based chemical reagents. Consequently, the search for specific chemical reagents has progressed remarkably, and to date, different strategies have been applied in activity-based probe (ABP) profiling, resulting in an increasing number of advanced and adequate reagents with broad utility in active enzyme detection, monitoring, imaging, and quantification.

In this review, a comprehensive description of the current methods for imaging serine proteases *in vitro* and *in vivo* with peptide-based reagents is provided. Fundamental concepts of ABP design and applications and emerging challenges and perspectives of current serine protease imaging reagents are discussed.

## Serine Proteases as Biomarkers for Personalized Treatment

**FIGURE 1:**
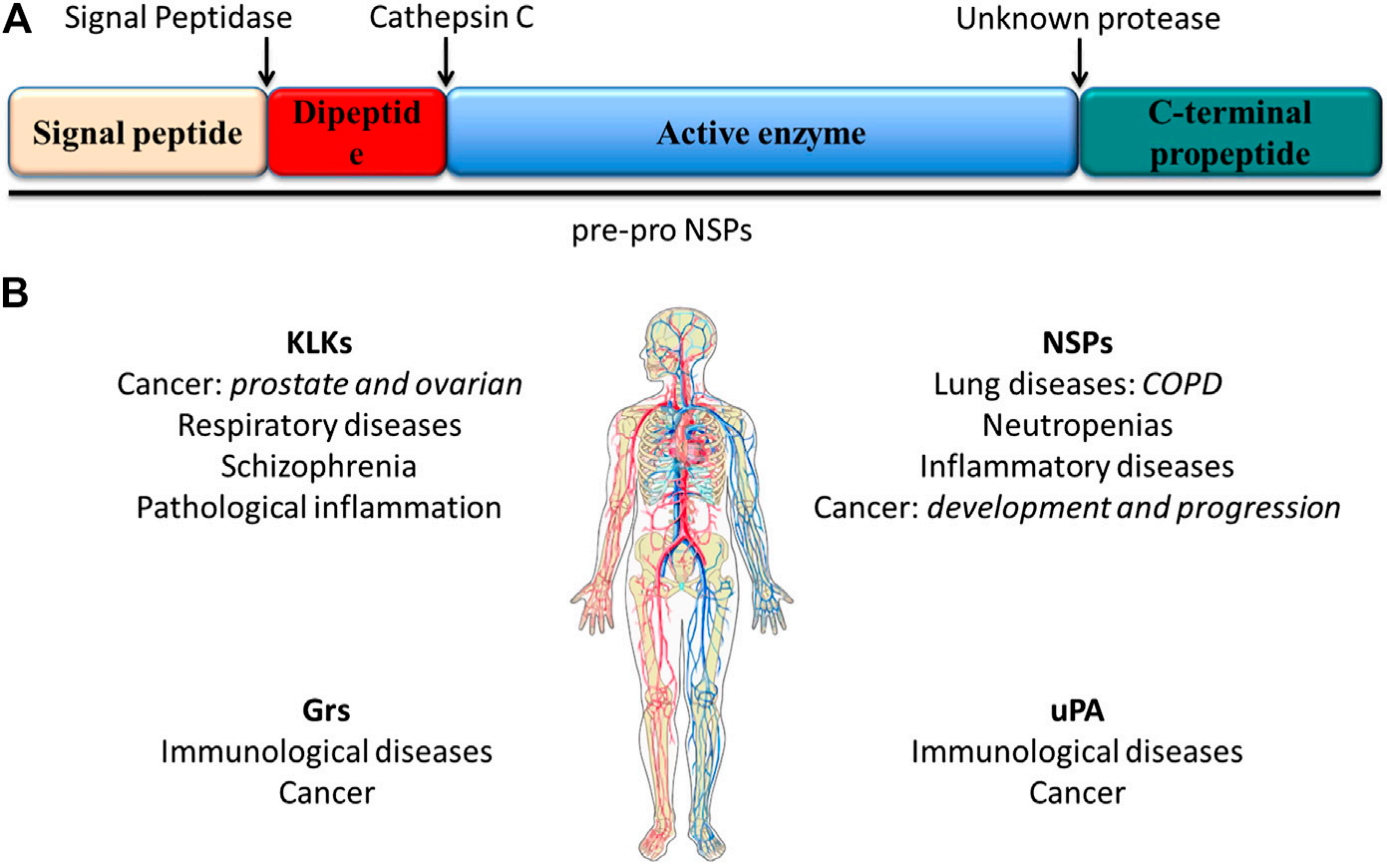
**(A)** scheme of the structural features of preproproteases that form the NSP family. These enzymes are expressed as preproenzymes typically containing a signal peptide removed by signal peptidases, a dipeptide removed by cathepsin **(C)**, a protease sequence and a C-terminal propeptide removed by an unknown mechanism. **(B)** Serine proteases and associated diseases. Uncontrolled active serine protease activity can cause the excessive hydrolysis observed in some diseases including lung diseases and cancer (Korkmaz et al., 2008; Korkmaz et al., 2010).

The serine protease group accounts for over 40% of all proteolytic enzymes in humans (Rawlings et al., 2002; Di Cera, 2009). Most of these enzymes are synthesized as preproenzymes, which are inactive zymogens, and require posttranslational processing to become functional. For example, NSPs are synthesized in the endoplasmic reticulum where their propeptide fragment is removed leading to conformational changes in the enzyme structure (Hedstrom, 2002; Korkmaz et al., 2008; Di Cera, 2009). Then, NSPs are transported to the granules and activated by mediating cathepsin C (CatC) hydrolysis of the prodipeptide (Pham and Ley, 1999; Korkmaz et al., 2008) (Figure 1A). The active serine proteases hydrolyze substrates with a canonical catalytic triad usually composed of three amino acids, namely, histidine-serine-aspartic acid, which facilitates the charge relay. For this, the hydroxyl group of catalytic serine attacks the carbonyl of the substrate peptide bond between P1-P1’, and after a series of events, the peptide bond is hydrolyzed.

Active serine proteases are abundant and are involved in different biological processess; thus, altered serine protease activity was found in blood coagulation system defects, such as thrombosis, and other diseases, including cancer. Therefore, serine protease inhibitors (serpins) are critical for the control and protection of organisms (Travis et al., 1990; Burgener et al., 2019). Serpins are functionally similar proteins that fold into a conserved structure and employ a unique suicide substrate-like inhibitory mechanism (Travis et al., 1990; Silverman et al., 2001). Consequently, only a small genetic mutation that can affect proper folding may disrupt protease inhibition. Thus, only recently have serine proteases and serpins been under investigation as diagnostic and prognostic biomarkers and potential drug targets (Docherty et al., 2003; Drag and Salvesen, 2010). This paragraph focuses on the importance of serine proteases as alternative biomarkers in personalized treatment and early diagnosis (Silverman et al., 2001) (Figure 1B).

### Neutrophil Serine Proteases as Potential Biomarkers

To eliminate pathogens, neutrophil granules are filled with a set of chemicals including active enzymes from the PA clan, mainly human neutrophil elastase (HNE), cathepsin G (CatG), proteinase 3 (PR3), and neutrophil serine protease 4 (NSP4), as well as less abundant Grs (GrA, GrB) (Wagner et al., 2004; Hochegger et al., 2007; Jena et al., 2012). Despite the similar mechanism of substrate hydrolysis, NSPs differ in substrate specificity, and thus, HNE and PR3 hydrolyze peptides after small aliphatic residues such as alanine (Ala) or valine (Val), GrA, and NSP4 after arginine (Arg), and GrB after aspartic acid (Asp), while CatG possesses dual substrate specificity and can hydrolyze peptides after Arg or phenylalanine (Phe) (Thornberry et al., 1997; Mahrus and Craik, 2005; Perera et al., 2012). This facilitates the broad functions NSPs play in the innate immune system. These enzymes are secreted from azurophil granules, in response to stimulation, to process interleukins, chemokines, and cytokines, but they can also hydrolyze a laminin and an elastin involved in matrix remodeling. Excessive NSP activity contributes to the pathogenesis of numerous disorders resulting from a massive inflammatory response, especially chronic inflammatory lung diseases or chronic obstructive pulmonary disease (COPD), type-1 diabetes and neutropenia (Horwitz et al., 2007; Zeidler et al., 2009; Korkmaz et al., 2010; Victor, 2016; Crisford et al., 2018).

COPD is the third leading cause of death worldwide and is characterized by chronic inflammation and the excessive infiltration of neutrophils into the airway lumen, where the release of serine proteases leads to major damage to connective tissue. Interestingly, in COPD, endogenous inhibitors fail to inhibit the activity of NSPs (Pandey et al., 2017; Crisford et al., 2018; McKelvey et al., 2020), but available preclinical and clinical data suggest that exogenous inhibitors suppress or attenuate the contribution of NSPs to the pathogenesis of lung diseases (Chughtai and O’Riordan, 2004; Voynow et al., 2008).

There are also diseases that are characterized by an abnormally low neutrophil count in peripheral blood, so-called neutropenia. The most frequently neutropenia is a result of autosomal dominant or autosomal recessive mutations (approximately 60%:40% of patients, accordingly) (Horwitz et al., 2007; Zeidler et al., 2009). The classic example of an autosomal dominant mutation is in the ELANE gene, which encodes HNE and affects the truncation of the NE C-terminus that potentially binds with adapter protein 3 (AP3) involved in the intracellular trafficking of NE to granules. This mutation may lead to the dislocation of NE, its uncontrolled activity and consequently damage to the organism (Nagy and Maquat, 1998). Further, based on 20°years of observation, both autosomal dominant or recessive mutations predispose patients to myelodysplastic syndromes or acute myeloid leukemia, with a probability of 25% for disease development (Miller et al., 1975; Corey et al., 1996; Boxer and Dale, 1997).

It is estimated that in the United States, severe cyclic and congenital neutropenia affect approximately 0.5–1 person per million people. Therefore, the interest in the personalized treatment and diagnosis of neutropenia and other NSP-related disorders is significantly growing. NSPs and biomarkers can be the future of personalized medicine and the early diagnosis of these diseases.

### Kallikreins

The kallikrein (KLKs) family is a serine protease family that includes 15 members (KLK-1–15) belonging to the PA clan. To date, numerous studies have demonstrated the physiological role of KLKs as regulatory proteases in signaling pathways and innate immunity, but they are also crucial for kidney and brain function (Kalinska et al., 2016). The vast majority of KLKs exhibit trypsin or chymotrypsin-like substrate specificity, with the exception of KLK-1, KLK-11, and KLK-14 that possess dual trypsin- and chymotrypsin-like preferences (KLK-2, KLK-4, KLK-5, KLK-6, KLK-8, KLK-10, KLK-12, KLK-13, and KLK-15 hydrolyze after the positively charged residues of Arg or lysine (Lys); KLK-3, KLK-7, and KLK-9 cleave peptide bonds after P1 tyrosine (Tyr), tryptophan (Trp) or Phe)) (Rawlings et al., 2002).

These proteases are expressed as preproproteins and are activated by trypsin-like cleavage after either Arg or Lys. Trypsin activates KLK-1, KLK-3, KLK-5, KLK-6, KLK-7, and KLK-15; thermolysin activates plasma KLKs; plasmin activates KLK-1; and lysyl endopeptidase activates KLK-6. The only exception is KLK-4, which is activated by metalloproteinase (Goettig et al., 2010; Kalinska et al., 2016). In homeostasis, the active KLKs are controlled by endogenous inhibitors such as serpins, macroglobulins and tissue serpins from the lympho-epithelial Kazal-type-related inhibitor (LEKTI) family and single metal ion inhibitors (e.g., Zn^2+^).

When all of the regulation steps fail, the excessive activity of KLKs is associated with several pathologies, including cancer, schizophrenia, respiratory diseases and pathological inflammation (Hong, 2014), but the best characterized is the role of KLK-3 in prostate cancer (Hong, 2014; Kalinska et al., 2016). Epithelial cells in the prostate almost exclusively produce prostate specific antigen (PSA), encoded by the KLK-related peptidase three gene. The circulating level of PSA increases in the presence of prostate cancer (Hong, 2014). Recently, other KLKs were also found in prostate cancer, and now they are under consideration as additional potential biomarkers. One of these enzymes is KLK4, for which a higher mRNA level correlates with a worse prognosis (higher score in the prostate cancer grading Gleason scale) and cancer stage. Among the other enzymes, KLK-14 and KLK-15 are associated with more aggressive tumors, while KLK-11 and KLK-5 are associated with a less advanced stage (lower Gleason score) (Hong, 2014).

Prostate cancer is the second-leading cause of cancer deaths for men in the United States, and it is estimated that approximately 10% of men will be diagnosed with this disease.

Despite the high abundance of serine proteases in humans and their activity associated with many diseases, they are rarely used as diagnostic markers. This may be due to the lack of precise chemical tools for investigating serine proteases, but considering the growing interest in this field, we can expect an increase in the importance of these enzymes in diagnosis in the near future, especially keeping in mind that PSA, the first protease used as a diagnostic biomarker, allows the fast and reliable diagnosis of prostate cancer with a simple blood test.

## Activity-Based Probes for Serine Protease Imaging

Our knowledge regarding serine proteases dramatically expands every day. However, despite the technological achievements, the majority of the research does not distinguish active enzymes and focuses on the total amount of these proteins that includes unprocessed, inhibited and active forms. Most recent studies have focused on antibody-related techniques that provide insights into the enzyme presence in cells; however, since active enzymes play significant roles in health and disease, it is imperative to study their activity rather than expression (Edgington et al., 2011). One of the often-used strategies to do so is the application of substrates to monitor proteolysis. However, none of the conventional techniques provide valuable information about the exact localization of active enzymes within cells or whole organisms.

**FIGURE 2:**
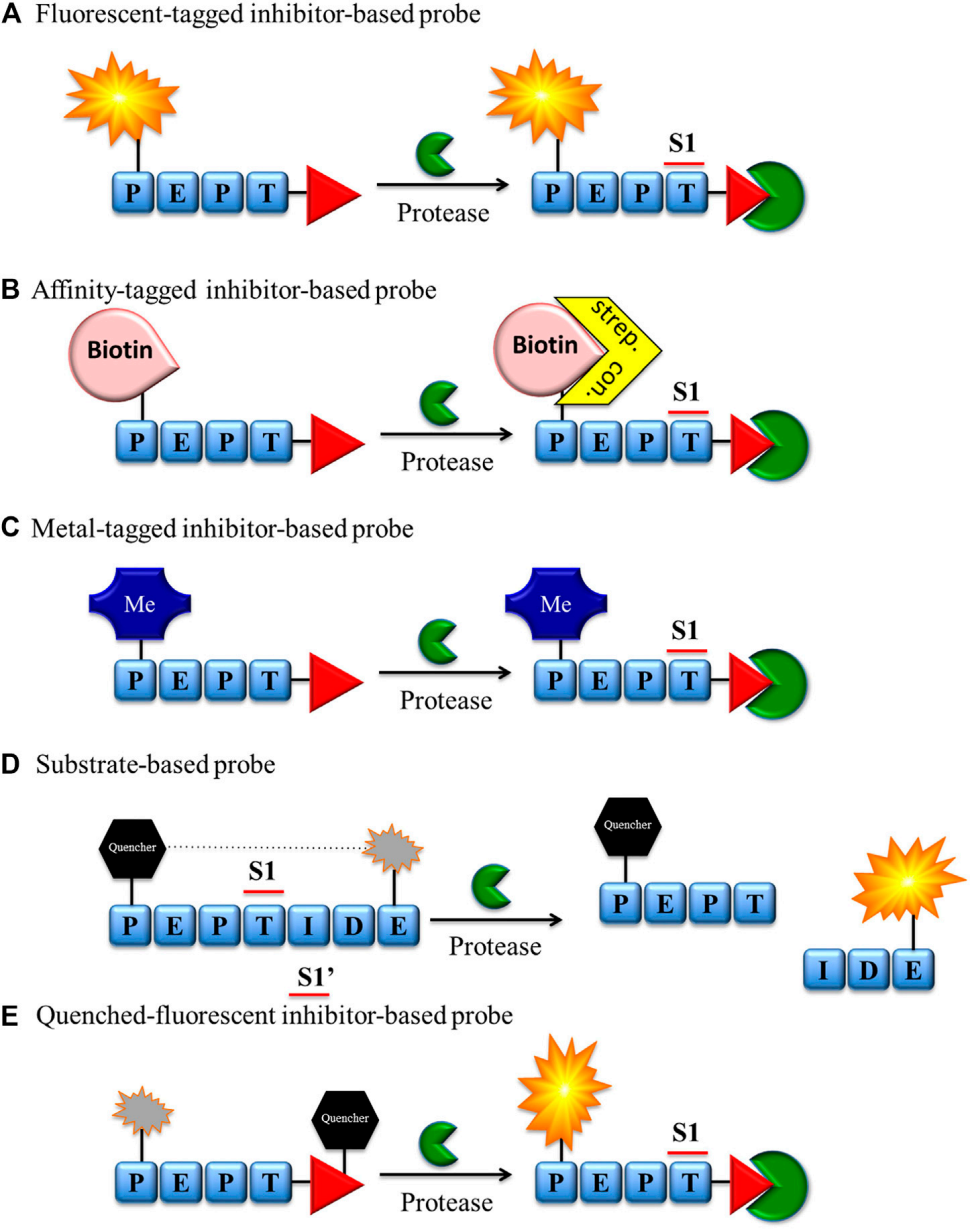
Chemical reagents for imaging serine proteases. **(A)** Fluorescent, **(B)** biotin, **(C)** metal-tagged and **(E)** quenched-fluorescent inhibitor-based bind covalently with the enzyme active site. The fluorescent probe-enzyme reactions **(A)**, **(D)**, and **(E)** can be detected after excitation of the sample with a fluorescence microscope or flow cytometry, while the biotin probe-enzyme complex **(B)** can be detected through biotin-binding protein (streptavidin or avidin) conjugated with a fluorescent label. **(C)** Metal-tagged probe-enzyme complex is directly detected using special equipment (e.g., CyTOF).

To circumvent this limitation, 3 decades ago, the first chemical molecule to discriminate active serine proteases was developed, allowing the imaging of serine peptidase (Woodard et al., 1994). At that time, these molecules were called biotinylated inhibitors, and notably, they were used not only for protein detection but also for its isolation and purification (Figure 2B). For some time, the development of serine protease imaging agents was neglected, but in parallel, investigators proposed many diverse strategies to dissect active cysteine proteases, which are reviewed elsewhere (Kato et al., 2005; Edgington et al., 2011; Deu et al., 2012). As inhibitors equipped with a fluorophore or biotin at the N-terminal end of specific peptide sequences (Figures 2A,B) dedicated to cysteine proteases are called ABPs, this nomenclature is now also widely used for serine proteases.

There are a number of technologies and strategies for active enzyme investigation and imaging, and new approaches are frequently proposed. Chemical reagents for serine protease imaging can be divided into two major groups based on their mechanism of interaction with targeted enzymes: substrate (Figure 2D) or inhibitor derivatives (Figures 2A–C,E). Both types of molecules have been applied in protease imaging, and each has its own strengths and weaknesses.

Classic ABPs (tag-peptide sequence-reactive group) are broadly used in the detection, imaging and analysis of serine proteases by SDS-PAGE, flow cytometry, mass cytometry, and fluorescence microscopy (Figures 2A–C). Despite the many advantages of these ABPs, the fluorescence of unbound probe is not quenched and, if not removed, can be equally detected. While it does not influence SDS-PAGE analysis, this may be troublesome in enzyme imaging or flow cytometry analysis. In these cases, to reduce the possibility of false positive readout, additional washing steps during the sample preparation are necessary, followed by careful analysis of the results.

To reduce the aforementioned issues, the Bogyo group developed a new smart strategy for cysteine proteases and equipped ABPs with a quencher moiety (qABPs) (Figure 2E) (Blum et al., 2007). In this technology, similar to classic ABPs, the protease is inhibited by the probe, but the fluorescence can be detected only after the probe binds with the enzyme. If the probe does not bind with the enzyme, the fluorescence is quenched, reducing the possibility of a false-positive signal. Although this strategy has been broadly applied for cysteine proteases, to date, the only probe for serine proteases of this type has been developed by the Verhelst group. As a reactive group, a mixed alkyl aryl phosphonate ester was applied for HNE and trypsin-like serine protease investigation (Serim et al., 2015) (Figure 2E). In this molecule, QSy7 is used as an acceptor of fluorescence to quench a signal from TAMRA (if excited). This proof of concept was successfully used for the gel detection of enzymes but has not yet been applied in the imaging of serine proteases.

Although the ABP and qABP have a wide range of applications in enzyme detection, they inhibit enzyme activity that may perturb normal cellular function, and this is the limiting factor in live imaging of these enzymes in cells. To address this issue, another solution of substrate-based molecules was proposed as a new type of reagent for protease imaging (Figure 2D). These reagents are built from a peptide sequence flanked with or without linkers and a fluorescence donor-acceptor pair with fluorophore-quencher groups (Edgington et al., 2011). These molecules do not bind covalently in the active site of the enzyme, and their fluorescence can be detected only after reaction with the targeted enzyme. Another advantage of substrate-based reagents is amplification of the fluorescent signal during readout, as uninhibited enzyme can hydrolyze many probe molecules, restricted only by the substrate turnover number and substrate concentration. These reagents possess many advantages, but in some circumstances, they may be troublesome. One of the main issues is the possibility of the diffusion of the peptide fragment containing the fluorophore not only from the hydrolysis milieu but also from the entire cell. To circumvent this issue, different probe modifications have been proposed, such as substrate lipidation, the addition of basic amino acids, or the application of fluorophores at the C-terminal end of probes (Hu et al., 2014; Janiszewski et al., 2020). The aforementioned strengths and weaknesses of chemical reagents for serine protease imaging should be considered in probe design and will be additionally discussed in the next section.

## Design of Serine Protease Imaging Reagents

Activity-based enzyme profiling is a relatively new branch of chemical biology; however, many aspects have been explored, and even more remain to be explored. Before designing protease imaging agents, it is crucial to plan their application and anticipated outcome. The cellular and *in vitro* properties of compounds characterized with good solubility, known selectivity, good potency, stability and reactivity, should be as well characterized.

**FIGURE 3:**
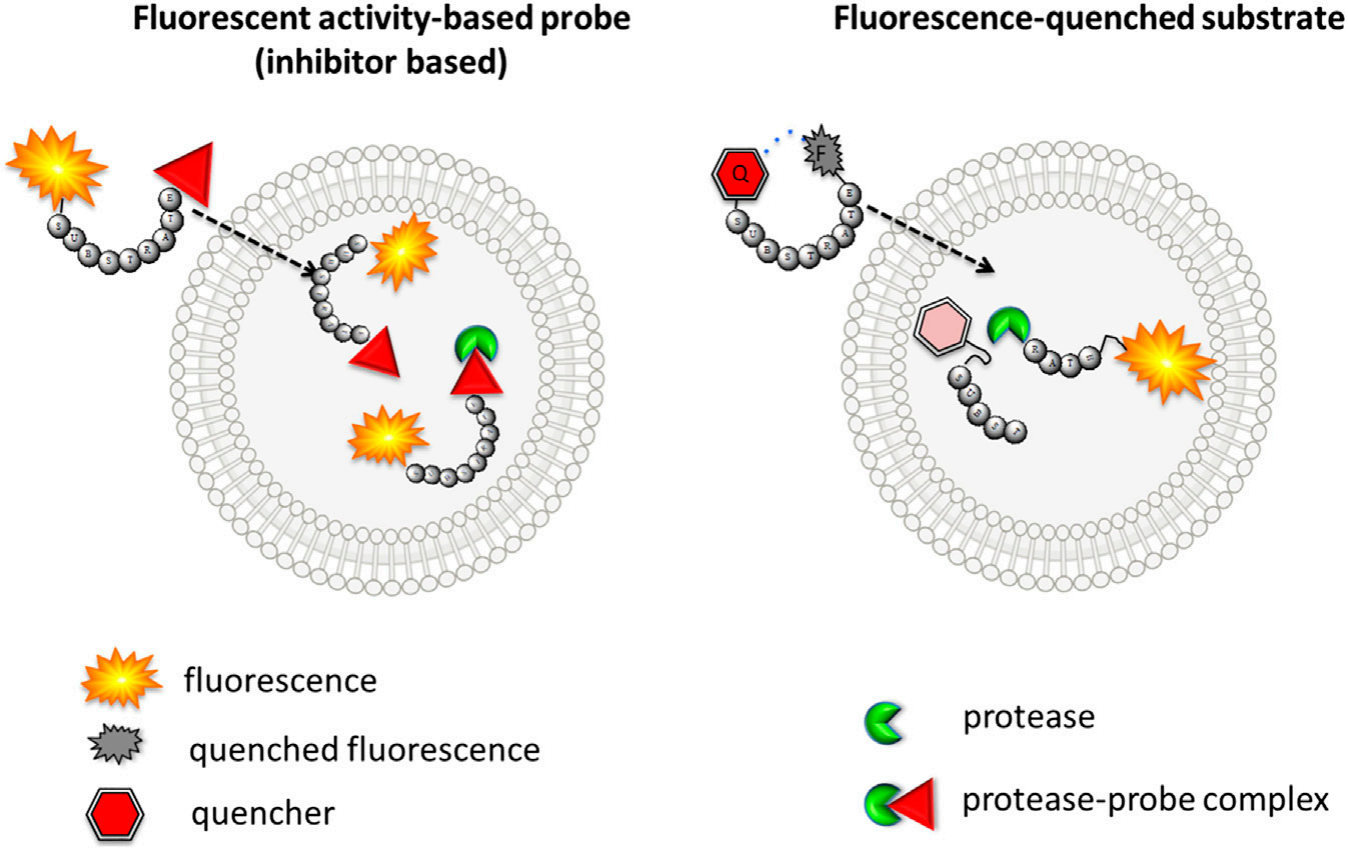
The scheme of the cellular uptake of fluorescence-quenched substrate and classic fluorescent ABP. Left—inhibitor-based fluorescent ABP is transported to the cell, and even fluorescence from unbound probe can be detected. Right–substrate-based probe fluorescence can be detected only if activated with the target protease.

ABPs with classic architecture (biotinylated, fluorescent, metal-conjugated, or quenched fluorescent) are built from the following three main elements: reactive group (warhead), specific sequence, linker(s) and fluorophores or fluorophor-quencher moieties (Figures 2A–C,E). They can be easily applied for quantification of the enzyme concentration and its detection in cellular lysates due to the presence of irreversible electrophilic reactive groups. These probes are also successfully used for enzyme imaging; however, if unbound/excessive probe is not removed, there is a possibility for a false-positive signal (Figure 3A). Unfortunately, this process is time consuming and needs to be evaluated for every cell type and individual probe separately. To reduce this inconvenience, it is recommended to apply the trace-labeling amount of probe that does not exceed the predicted enzyme concentration.

The other type of chemical reagent is built from the substrate sequence flanked with fluorescence donor-acceptor pairs. These probes are more suitable for imaging and can even be applied in enzyme observation in real time; however, they are not useful for enzyme in-gel detection or enzyme quantification (Figure 3B). Both substrate- and inhibitor-like reagents can be applied in serine protease detection in cells as well as in whole organisms (Table 1). This paragraph focuses on the design of imaging reagents for serine proteases and provides principles for the selection of their building blocks.

**TABLE 1:** Strengths and weaknesses of different types of serine protease probes described within the text.

	Biotinylated inhibitor-like probes	Fluorescent inhibitor-like probes	Metal-tagged inhibitor-like probes	Quenched fluorescent activity-based probes	Substrate-like probes
Covalent binding	YES	YES	YES	YES	NO
Quantification	YES	YES	YES	YES	NO
Fixed sample imaging	YES	YES	YES	YES	YES
Live imaging	NO	YES^a^/NO	NO	YES	YES
Direct in-gel detection	NO	YES	NO	YES	NO
Western blot	YES	YES	YES	YES	NO
Flow cytometry	YES	YES	NO	YES	YES
Mass cytometry by time-of-flight	NO	NO	YES	NO	NO
Fluorescent microscopy	YES	YES	NO	YES	YES
Enzyme isolation	YES	NO	NO	NO	NO
Low risk of diffusion	YES	YES	YES	YES	NO
*In vitro* application	YES	YES	YES	YES	YES
*In vivo* application	NO	YES	NO	NT^b^	YES
Influence on cell function	YES	YES	YES	YES	NO

a Only under certain conditions.

b NT—not tested but possible.

### Reactive Group Selection for Serine Protease Imaging Reagents

**FIGURE 4:**
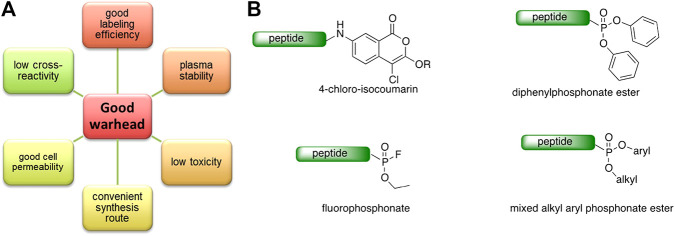
**(A)** Properties of a good warhead. **(B)** Chemical structures of electrophiles applied in ABPs for imaging serine proteases: 4-chloro-isocoumarin, diphenylphosphonate, FP and mixed alkyl aryl phosphonate ester.

The main function of reactive groups is to covalently attach the probe to the target enzyme that prevent diffusion of the probe-enzyme complex. While selecting an adequate reactive group, its properties, such as its labeling efficiency, cell permeability, cross-reactivity with other proteases and stability, need to be evaluated. Reactive groups that are currently applied for serine protease imaging agents are electrophiles that irreversibly bind with the active site nucleophile, a hydroxyl group of the catalytic serine residue (Powers et al., 2002) including the following: fluorophosphonates (FPs), and Table 2 *α*-amino-alkyl diphenyl phosphonates, 4-chloro-isocoumarins, and mixed alkyl-aryl phosphonate esters (Figures 4A,B).

**TABLE 2:** Serine protease reactive groups and their characteristics.

Warhead scaffold	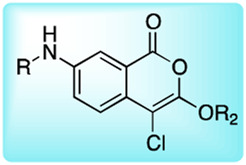	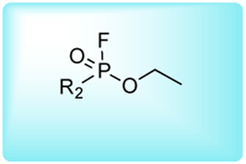	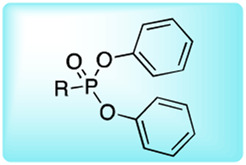	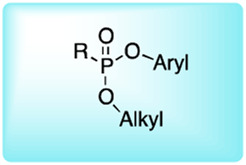
Name	4-Chloroisocoumarin	Fluorophosphonate	Diphenyl phosphonate	Alkyl aryl phosphonate
Stability in buffers	High	Medium	High	High
Stability in plasma	Low	Medium	High	^a^ND
Selectivity	Serine proteases (cysteine proteases)	Serine hydrolases	Serine proteases	Serine proteases
Reactivity	Moderate	High	High	Medium
Synthesis	Easy	Difficult	Easy	Difficult

a No data available.

**FIGURE 5:**
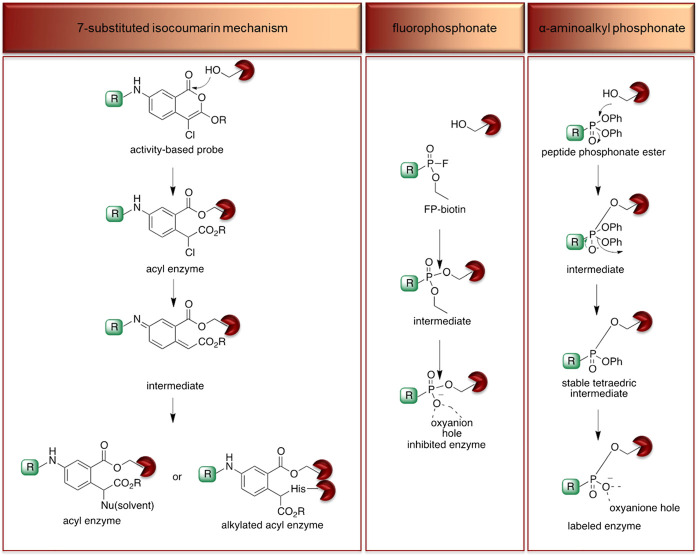
Proposed mechanism of serine protease active site inhibition by different serine protease reactive groups.

The isocoumarin warhead was incorporated in one of the first biotinylated ABPs for serine protease for lymphocyte granzyme detection (Kam et al., 1993). These reactive groups are heterocyclic irreversible inhibitors of serine proteases. The active site serine residue opens the isocoumarin ring, forming a stable acyl enzyme derivative (Figure 5) (Kam et al., 1993). While isocoumarins have been broadly used as inhibitors of serine protease, in ABPs, this reactive group has been applied only occasionally, as some derivatives have been found to unspecifically inhibit cysteine proteases (Powers et al., 2002). In addition, isocoumarins have limited stability in plasma and high stability only in buffers; therefore, their utility is limited to basic biochemical assays (Powers et al., 2002).

The other reactive group, phosphonyl fluoride (FP), was described as one of the first irreversible inhibitors of serine proteases and has been known for approximately 100 years. The FP warhead has been applied in the serine protease ABP, resulting in the synthesis of an FP-biotin probe with high sensitivity and broad specificity to all serine hydrolases (Liu et al., 1999). There are only a few ABPs with the FP warhead flanked with tetramethylrhodamine, fluorescein or biotin as detection tags (Kidd et al., 2001; Patricelli et al., 2001). The irreversible binding of FP with proteases is determined by the electrophilicity of the phosphorus atom. The mechanism of stable enzyme-inhibitor complex formation probably occurs by the replacement of the fluoride group on the phosphorus atom with a nucleophilic serine hydroxyl group in the active site (Figure 5). The presence of a fluoride atom in phosphonyl fluorides significantly increases the reactivity, but it led to the high toxicity exemplified by rapid binding with acetylcholinesterase (Powers et al., 2002).

Despite the many advantages of FP as a warhead, there has been a shift from using it in ABPs to using α-aminoalkyl phosphonates, which are now the most frequently used reactive group for serine endopeptidase ABPs. α-Aminoalkyl phosphonates were developed by Oleksyszyn et al. (Oleksyszyn and Powers, 1991) as specific to serine proteases. This type of warhead constitutes a compromise between activity and chemical stability since the phenoxy group is less electronegative than the fluorine atom but is sufficient for the reaction with nucleophilic hydroxyl groups on the active site serine residue (Powers et al., 2002). The mechanism of inhibition is similar to that of FPs; the active site serine hydroxyl group attacks the phosphorus atom, and a pentacoordinate intermediate is formed. Next, one of the phenoxyl groups leaves and simultaneously forms a tetravalent phosphonylated derivative, maintaining the second phenoxyl group that is next removed during the so-called aging process. The formed serine phosphomonoester is stabilized by the interactions of phosphonate oxygens in the oxyanion hole (Figure 5).

The application of α-aminoalkyl phosphonate in ABP has a few advantages, and the most important is the exclusive binding with serine proteases. The majority of ABPs incorporating α-aminoalkyl phosphonates flanked with peptide sequences do not react with acetylcholinesterase and are chemically stable in plasma (Powers et al., 2002). Furthermore, these compounds are characterized by high stability in buffer solutions as well as in human plasma, making them more suitable for biological analyses (Oleksyszyn and Powers, 1991). Their additional advantage is the susceptibility of the ester groups to modifications that can increase the selectivity and potency (Sienczyk et al., 2011).

In the majority of approaches for serine protease probes incorporating α-aminoalkyl phosphonates, both ester groups are modified, but interestingly, in one study, the Verhelst group proposed a replacement of one ester group with a quencher molecule (Serim et al., 2015) leading to asymmetry on the phosphorus group. Although this modification leads to a mixture of diastereomers being obtained, it does not significantly change the specificity and reactivity of the ABP but allows the specific detection of the target enzyme. By fluorescence quenching, the false-positive signal is reduced, maintaining the covalent binding of the ABP with the hydroxyl group of the catalytic serine. The above arguments support the hypothesis that the α-aminoalkyl phosphonate ABP can be suitable for *in vivo* applications, but prior to application, it needs to be well validated. For example, it was found that the phosphonate probe designed for the irreversible binding of urokinase-type plasminogen activator (uPA) also binds KLK-4 and KLK-8 but in a reversible manner. This demonstrates that even careful warhead design does not guarantee the anticipated outcome (Vangestel et al., 2016).

### Serine Protease Recognition Sequence Selection—Chemical Methods

The key fragment of the ABP is a recognition sequence that allows specific binding with target proteases or a group of proteases. The first ABP studies involved a marker that lacked specificity and labeled all serine proteases All serine proteases recognize, bind and hydrolyze peptides in the active site cleft using the same mechanism, but the shape surrounding the active site differs. This area is formed by so-called pockets, three-dimensional shapes in the enzyme structure that are unique for every protease (also called a fingerprint) (Schechter and Berger, 1967). The chemical character of these pockets guarantees enzyme specificity and allows the selective hydrolysis of endogenous substrates. This feature is one of the key factors in the design of ABPs for the successful detection of the target enzyme. The search for leading sequences is challenging; however, frequently, peptides or other linear linkers are successfully incorporated.

**FIGURE 6:**
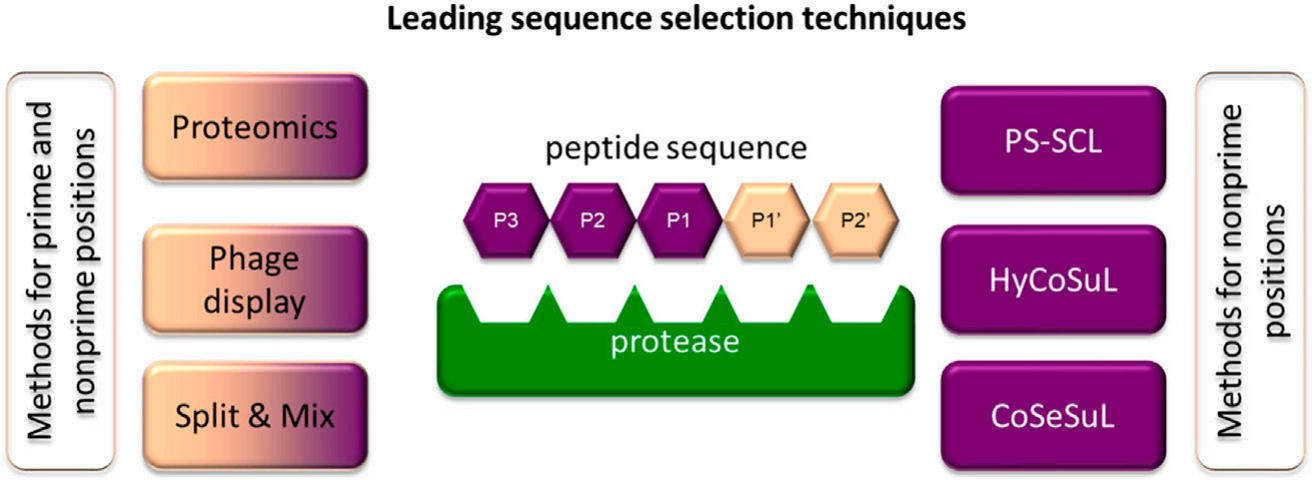
Methods frequently applied to identify specific sequences for chemical reagents for serine protease imaging. Some of the techniques can be applied to investigate prime and nonprime positions.

Recently, there has been a rapid technological leap in the search for the substrate specificity of proteases. In this review, only the most frequently used chemical methods for specificity profiling are briefly discussed, while all technologies for determining the substrate specificity are discussed elsewhere (Faucher et al., 2020; Figure 6).

One of the most frequently utilized chemical methods for determining substrate specificity is the positional scanning-synthetic combinatorial library (PS-SCL) (Harris et al., 2000). This approach for incorporating isokinetic mixtures in peptide synthesis was proposed in 1994 by Ostresh and coworkers and until recently was one of the most frequently used methods (Ostresh et al., 1994). The greatest advantage of this technology is the use of solid-phase peptide synthesis (SPPS) in combinatorial chemistry. For example, the substrate library for investigating the S2 pocket consists of the defined and selected amino acid to be recognized by the target enzyme at the P1 position, isokinetic mixture in the P3 and P4 positions and individual amino acids in the P2 position. As a fluorogenic residue, three coumarin derivatives frequently differ in the substitution in the 4^th^ position (coumarin nomenclature is used): 7-amino-4- (trifluoromethyl)coumarin (AFC) (Cheng et al., 2002), 7-amino-4-methylcoumarin (AMC) (Zimmerman et al., 1977) or 7-amino-4-(carbamoylmethyl)coumarin (ACC) (Harris et al., 2000). However, due to the bifunctional character of ACC (two reactive groups—amine and carboxyl), it has the broadest application. The amino-conjugated ACC substrate cleavage by target enzyme leads to the release of free ACC and the shift of the excitation and the emission maxima from 325 to 400 nm to 350 and 450 nm respectively. When peptide hydrolysis occurs between P1 and the fluorophore, the amine group of ACC deprotected, and fluorescence can be detected at 450 nm (Harris et al., 2000). The hydrolysis rate can be easily observed using spectroscopy by monitoring of the fluorescence signal increase over time, as it is proportional to hydrolysis of the substrate. This allows the fast prediction of catalytic preferences. From all of the residues, the best sequences for achieving activity, selectivity and solubility are selected as optimal substrates for the enzyme of interest. This strategy was used for the optimization of leading sequences for both cysteine and serine proteases and is repeatedly applied in ABPs (Rano et al., 1997; Thornberry et al., 1997). Recently, this strategy was improved by the incorporation of a set of unnatural amino acids that allowed for more adequate screening of enzyme catalytic preferences and better accommodation of substrates in enzyme pockets, leading to an increase in their specificity and potency (Kasperkiewicz et al., 2014). This strategy is called Hybrid Combinatorial Substrate Library (HyCoSuL), and to date, it has been used to distinguish between closely related enzymes belonging to the same family of serine proteases (NE (Kasperkiewicz et al., 2014), CatG (Kasperkiewicz et al., 2017), PR3 (Kasperkiewicz et al., 2017), NSP4 (Kasperkiewicz et al., 2017; (Kasperkiewicz et al., 2015), GrA (Kolt et al., 2020), and GrB (Janiszewski et al., 2020)) and cysteine proteases (caspases (Poreba et al., 2015; Poreba et al., 2019) and cathepsins (Poreba et al., 2019; Poreba et al., 2018)).

Both the PS-SCL and HyCoSuL strategies represent a technological leap and have advanced the study of ABPs for serine proteases. However, these strategies allow for the optimization of nonprime positions of the substrate sequence, while prime positions are equally important, especially in substrate-like probes. To study prime position specificity, internally quenched fluorescence (IQF) substrates, a branch of FRET (Forster resonance energy transfer) libraries, are used (Oliveira et al., 2012; Yaron et al., 1979). It is worth noting that they can also be applied to investigate nonprime pockets. In this type of library, nonprime and prime positions can either be defined as an amino acid or an isokinetic mixture of amino acids. Importantly, only one of the positions, the one investigated, is variable, while the others remain fixed. The peptide sequence is flanked by a fluorescence donor and acceptor pair that is characterized by overlapping fluorescence donor emission with fluorescence acceptor excitation, enabling fluorescence quenching. After substrate hydrolysis by the investigated enzyme, the distance between the donor and acceptor increases, leading the molecule to fluoresce when excited, which can be monitored spectroscopically. In a search for specific sequences, in this strategy, frequently used fluorophore-quencher pairs are Edans-Dabcyl (Maggiora et al., 1992), ABz-Tyr (NO_2_) (Wysocka et al., 2014), ABz-EDDNP (Oliveira et al., 2012), MCA-Lys (DNP), and ACC-Lys (DNP) (Poreba et al., 2017).

The other chemical approach incorporating combinatorial chemistry, called the “split and mix” technique proposed by Furka et al., is suitable for determining both prime and nonprime pocket catalytic preferences. In this technique, to obtain an equimolar mixture of amino acids after the attachment of the first amino acid to the resin, it is split into equal portions. To these portions, additional amino acids (different in every portion) are attached, the portions are mixed and split again, and the peptide is elongated. This procedure is repeated until the required peptide length is reached (Furka et al., 1991). As in PS-SCL and HyCoSuL, the peptide sequence is flanked with the fluorophore (for prime and nonprime pockets) or fluorescence donor-acceptor pairs (for nonprime pockets). With this strategy, prime sites were investigated for a set of serine proteases, including CatG (Groborz et al., 2019) and PR3 (Popow-Stellmaszyk et al., 2013).

The “split and mix” technique and technologies incorporating isokinetic mixtures have their supporters and opponents; however, both strategies allow the rapid analysis of hundreds of compounds in parallel. It is worth noting that screenings are only a first step and always need to be validated to select optimal leading sequences. These strategies revealed that it is possible to choose an ABP recognition sequence that is specific for a single protease, but it is always a challenging process.

### The Selection of Linkers and Additional Peptide Sequence Modifications

The recognition sequence of biomarkers is usually flanked by a linker(s) that function(s) as spacers of the reactive fragment from a tag and to reduce tag binding in the enzyme active site that might influence the probe specificity. As linkers, amino acids such as simple aliphatic glycine or linear derivatives such as β-alanine are used. The other type of linkers are long alkyls including commonly used 6-aminohexanoic acid (6-Ahx or Aca). This type of linker has been successfully applied in ABP for some serine proteases, including CatG, PR3, NSP4, GrA, and GrB (Kasperkiewicz et al., 2014; Kasperkiewicz et al., 2015; Kasperkiewicz et al., 2017; Janiszewski et al., 2020; Kolt et al., 2020). Despite the broad utility of 6-Ahx, this linker is hydrophobic and to compensate for the polarity it is recommended for reagents with more hydrophilic peptide part, while for more hydrophobic one, polyethers are a better choice. These linkers are equipped with additional oxygen groups in the structure increasing reporter solubility and permeability throughout the cell membrane. The classic examples of polyethylene glycols (PEGs) are PEG (4) and PEG (2), which have been applied in HNE and CatG probes (Gehrig et al., 2012; Kasperkiewicz et al., 2014; Guerra et al., 2019).

Aforementioned linker residues are frequently used in the probe structure; however, in some cases they can be replaced with neutral peptides. These peptides may moderate the solubility and cell permeability, but they may also influence the potency of the probe. Therefore, to reduce the negative influence of longer linkers, usually the peptide chain length is optimized. This type of optimization was performed for example for GrB (Janiszewski et al., 2020), where the more distant peptide-recognizing pockets, possessing broader specificity and recognizing a vast majority of amino acids, influence the kinetic parameters of enzyme-probe binding.

The probe sequence modifications may play a variety of functions and one of them is the improvement of signal accumulation in substrate-based markers. In these reagents, the signal can be detected after cleavage by the enzyme and, ideally, the fluorophore should accumulate where hydrolysis occurs. However, Hu et al. found fluorophore diffusion when using such molecules for cathepsin S (CatS) imaging. To reduce this obstacle, different strategies can be considered, but one of the most frequently used is probe lipidation by the addition of a lipid anchor that binds with the outer leaflet of the plasma membrane to the peptide sequence limiting the diffusion of the hydrolysis product (Hu et al., 2014). The anchoring of the hydrolysis product is not the only advantage of probe lipidation. This strategy has also been used to monitor protease activity at the cell surface (CatG) (Guerra et al., 2019) or membrane-bound enzymes (PR3). In another strategy, the fluorophore can be attached to the C-terminal end of the probe while the quencher is attached at the N-terminus. After hydrolysis, the newly formed ^+^H_3_N-peptide-fluorophore fragment bears a positive charge that reduces diffusion, as was demonstrated by Janiszewski et al. (Janiszewski et al., 2020).

### The Selection of Tags

While the recognition sequence and reactive groups are crucial for enzyme reaction with an ABP or substrates, tags are essential for their detection. The main advantage of these chemical reagents when compared with immunoassays is their easy and straightforward application to the cells, which can be directly followed by analysis. For this, tags should be selected depending on the planned application (*in vivo* or *in vitro*, in-gel, microscopically, or in the whole body) and available analytical equipment. Hence, the most frequent groups of tags currently used as chemical markers for serine proteases are reviewed below.

*Radioisotopes:* The first tags applied in ABPs were radioisotopes (Mason et al., 1989). The main advantage of their utility is their low price, high sensitivity and low background during readout. They can be detected with autoradiography, positron emission tomography (PET) (Ides et al., 2014) or single photon emission computed tomography imaging (Vangestel et al., 2016). Despite many advantages, the utility of radioisotopes in ABPs possesses many drawbacks, including a short half-life (low stability). Due to the short period of radiation, their utility requires special safety procedures because of the radiation, making them less convenient to use (Yuan et al., 2006). Classic examples of radioisotopes used in ABPs are ^125^I, ^3^H, ^18^F, and ^111^In. Tritium (^3^H) at the N-terminus do not influence the ABP structure; however, its detection is low, making sample exposition time consuming and leading to difficult analysis of the results (Fenteany et al., 1995). On the other hand, ^18^F ABPs demonstrate better detection but possess low tumor uptake, slow blood clearance, and low stability (Ides et al., 2014). Alternatively, ^111^In with a half-life of 2.80°days can be applied. For example, in one of the ABPs, the 1,4,7,10-tetraazacyclododecane-1,4,7,10-tetraacetic acid (DOTA) was complexed with ^111^In, indicating a similar inactivation rate potency, higher stability and improved cellular uptake when compared with the probe containing^18^F (Vangestel et al., 2016).

*Affinity tags:* Affinity tags are the second group of reporters applied in ABPs. A classic example of a frequently applied molecule is biotin. This moiety is often attached to the peptide sequence and can be indirectly detected with biotin-binding protein (streptavidin or avidin) conjugated with fluorescent label (Kam et al., 1993; Kasperkiewicz et al., 2014), as the biotin binding affinity of streptavidin is one of the highest in biological systems. Interestingly, ABPs armed with biotin can be applied not only for active enzyme detection and its imaging but also for protein isolation and the proteomic identification of active proteases. The main strength of biotinylated probes is their versatility, as a selection of fluorophores can be attached to streptavidin, making it feasible for almost every type of equipment. Furthermore, biotinylated ABPs are relatively inexpensive, straightforward in synthesis and safe to use. Despite all of these advantages, the application of biotinylated ABPs requires numerous steps that increase the analysis time. A disadvantage of fluorescent streptavidin conjugates is that they may attach to naturally occurring biotinylated proteins in lysates or cells, causing a false-positive readout (Sadaghiani et al., 2007), but these probes are still one of the most reliable and therefore frequently used probes.

The bulky reporter groups possess low cell membrane permeability and can hinder the cellular uptake of ABPs. Hence, to address this limitation, azides and alkynes were used and found to be more advantageous (Sadaghiani et al., 2007). They react with one another in a copper-catalyzed reaction and form triazole products in a so-called click reaction (Speers and Cravatt, 2004). In one of the examples, it was demonstrated that tag-free alkyne-FP labels serine proteases with greater affinity than analogous FP probes armed with biotin, and moreover, this probe has better cell permeability. However, despite many promises, the aforementioned strategy was applied only a few times in ABPs for serine protease detection, as it is not the most convenient strategy and is not recommended in live cell imaging.

Fluorophores: Recently, there has been increasing interest in fluorophore applications in ABP structures. The use of fluorophores facilitates the detection of targeted serine proteases, and these tags are safer and more straightforward to use than radioisotopes or affinity tags. Although a broad repertoire of fluorophores is already in use, new and improved moieties are still being developed. Only recently was the vast selection of fluorophores extended with molecules characterized by different emission/extinction wavelengths, varying brightness, photobleaching and stability (Heal et al., 2011).

**FIGURE 7:**
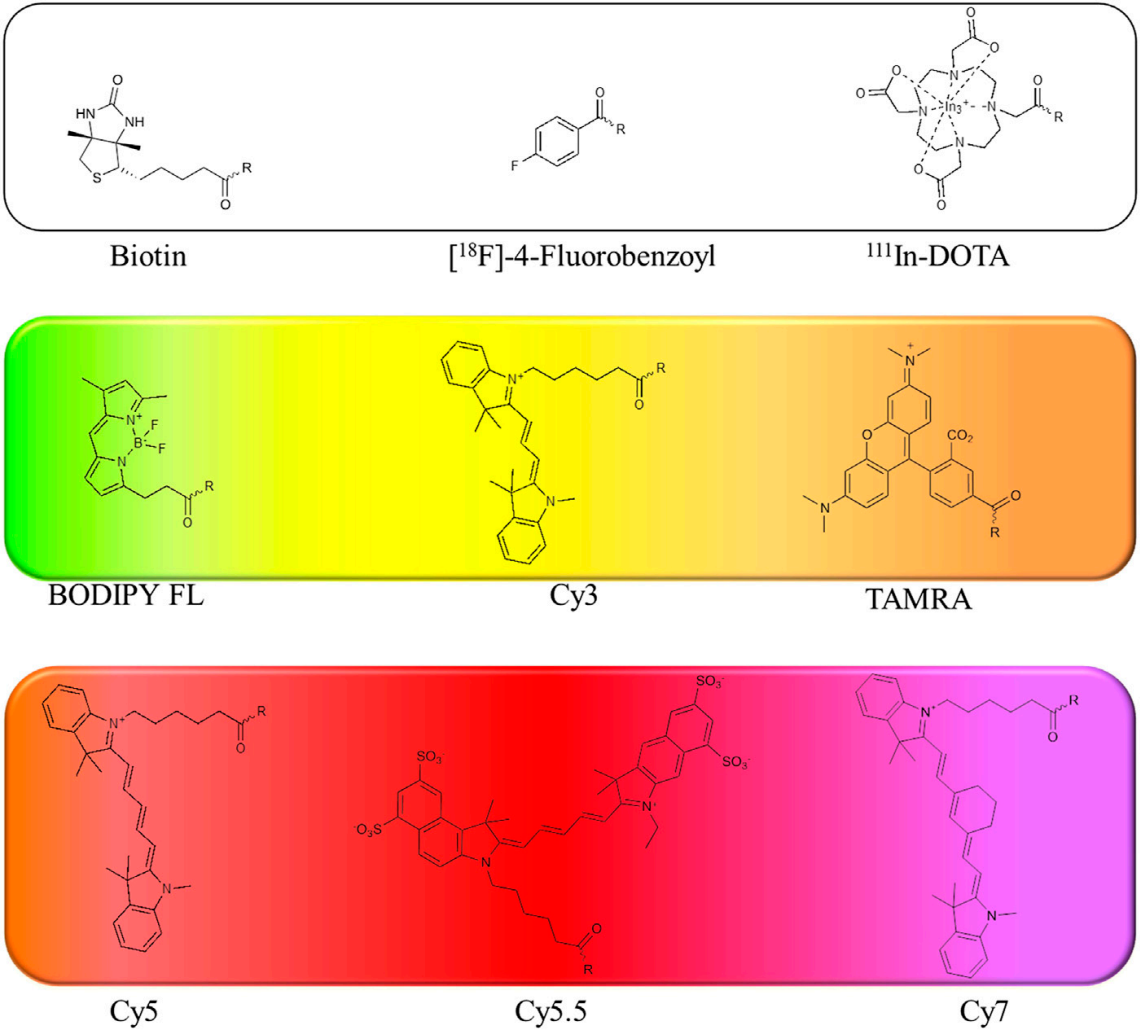
Examples of affinity, fluorescent and metal tags applied in the imaging of serine proteases.

Several of the first fluorophores used in ABPs were fluorescein and rhodamine, which are inexpensive, safe and straightforward to use in synthesis and detection. However, their high photobleaching and very low cell membrane permeability reduce their utility. Therefore, fluorescein has been replaced with BODIPY^FL^, while rhodamine has been replaced with cyanine 3 (Cy3), which are more resistant to photobleaching and possess higher brightness (Figure 7).

When selecting a perfect fluorophore, it is crucial to remember that its emission should not overlap with the cell autofluorescence; therefore, where the possible background is a concern, moieties characterized with higher excitation and emission spectra, such as cyanine derivatives, namely, cyanine 5 (Cy5) and cyanine 7 (Cy7) as well as their derivatives, are used due to their far-red and near-infrared (NIR) emission. In addition, the NIR spectrum provides maximal tissue penetration and minimal absorption by physiological absorbers such as hemoglobin or water. The other advantages of these cyanine derivatives in chemical reagents for live cell imaging include their stability in cell culture media, high brightness and low photobleaching. Despite their many advantages, classic cyanine derivatives (Cy3, Cy5, and Cy7) are characterized by high hydrophobicity and low cellular penetration. Facing this problem, different cyanine modifications are available, including sulfo, methyl and carboxyl derivatives. Cyanines seem to be the fluorophores of choice for chemical reagents for active enzymes, allowing rapid enzyme detection in lysates using SDS-PAGE as well as for enzyme imaging in cells and even whole organisms.

Further, fluorescent ABPs allow the parallel imaging of a couple of enzymes, allowing the simultaneous observation of the target proteins in the same cell (Berkers et al., 2005) by analysis of a few ABPs with distinct excitation/emission wavelengths in the same sample. These parameters may be analyzed by flow cytometry, with a limit of approximately 19 parameters, or microscopically, with approximately 5 parameters at the same time.

*Lanthanides:* Lanthanides are the last group of metal tags found in chemical reagents for active enzyme detection and imaging (Figure 2). One of the first approaches incorporating lanthanides for the investigation of serine proteases dates back to 2013, when the enzyme activity was detected with CatalyCEST magnetic resonance imaging (MRI) by monitoring substrate-like reagents containing Tm-DOTA (Yoo et al., 2014). Later, another attempt in the search for a perfect solution in serine protease imaging was made by the broad application of lanthanides in inhibitor-like probes. In this strategy, lanthanides were attached to the linker, similar to fluorophores, biotin or radioisotopes, and the labeled enzymes were detected with cytometry by time-of-flight (CyTOF) (Poreba et al., 2020). Similar to ABPs with fluorophores or biotin, lanthanide probes can also be imaged; however, the resolution of the images is lower than that in classic microscopic analyses. The careful experimental design allows parallel analysis of enzymes with other cellular components in a single cell due to the differences in the molecular weights of individual metals. As in the case of fluorescent probes, excessive amounts need to be removed to provide reliable results, as unbound probe may provide a false-positive signal. The other disadvantage of this strategy is the high price of reagents and equipment.

### Donor-Acceptor Pair Selection

In substrate-based probes or qABPs, for the efficient self-quenching of fluorescence that remains optically silent in the nonactivated state, the fluorescence donor and acceptor are placed in close enough proximity to each other and are distanced with a peptide sequence (Serim et al., 2015). In the case of chemical reagents for serine protease imaging, fluorescent quenching occurs via a FRET mechanism, where the fluorescence donor and acceptor are distanced with a specific sequence. An appropriate donor and acceptor pair can be selected based on the spectral overlap between the emission of the donor and the absorption of the acceptor. To obtain the required effect, two main types of fluorescence donor-acceptor pairs are currently used. The first type is two fluorophores, and the second type is a fluorophore and nonfluorescent molecule, called a quencher.

There are a few examples of donor-acceptor pairs that consist of two fluorophores, The energy transfer takes place between these fluorophores and one of them excites the other one, as for example TAMRA-coumarin 343 pair. In this example of FRET transfer, the hydrolysis of peptide sequence leads to a difference in fluorescence emission; an approximately 8.9-fold increase in donor emission and a 0.9-fold decrease in acceptor fluorescence. The exact characterization of the TAMRA-coumarin 343 pair leads to the conclusion that this donor-acceptor pair can be successfully applied to basic diagnostic applications, as it has been used to monitor CatG activity over time (Guerra et al., 2019). When two fluorophores are used, they both contribute to an overall background due to their fluorescent nature. This problem can be reduced by the application of the second donor-acceptor pair type, where the used acceptor is not intrinsically fluorescent and the transferred energy is released in nonradiative processes such as molecular vibration or heat; therefore, it is called a dark quencher (turn-on fluorescence). The most frequently used dark quencher is 4-((4-(dimethylamino)phenyl)azo)benzoic acid (DABCYL), but its absorption spectrum overlaps with reporter dyes emitting only below 480 nm. Black hole quenchers, namely, BHQ1, BHQ2, and BHQ3, absorb at 534, 579, and 672 nm, respectively, and can address this limitation; for example, the Cy3-BHQ2 pair was applied in the labeling of GrB (Janiszewski et al., 2020). In another example, TAMRA was quenched by QSy7, another nonfluorescent acceptor (Serim et al., 2015). Donor-acceptor fluorescence pairs that might be applied in future ABPs for serine proteases are described elsewhere and are used for oligonucleotides (Marras et al., 2002).

## Peptide-based Strategies for Imaging Serine Proteases

Surprisingly, not all of the enzyme detection agents used for enzyme detection are applied for active enzyme imaging. There are many examples of very useful chemical probes to study the function and biochemistry of enzymes, and this section focuses on chemical agents successfully applied for the imaging of active serine proteases.

The first attempts in enzyme activity imaging were made in 1994 by the Powers group who used the newly developed peptidyl α-aminoalkyl phosphonate inhibitor labeled with fluorescein (FTC) [FTC-Aca-Ala-Ala-Met^P^(OPh)_2_] to label enzymes in RNK-16 cells. This ABP reacted with distinct, granule-like regions of the natural killer cell line, RNK-16, suggesting granzyme labeling (Abuelyaman et al., 1994). With this probe, the authors demonstrated a proof of concept of active enzyme imaging, which is constantly being improved.

The most frequently imaged serine proteases are neutrophil serine proteases, especially HNE, PR3 and CatG. This is probably due to their intracellular presence and high concentration in primary granules. Elastase substrates, inhibitors and probes have been investigated for decades, but despite many efforts, their imaging is not trivial. In 2011, the Peterson group developed an HNE-specific NIR fluorescence imaging agent (NE680) that is a substrate-based probe with a sequence dedicated to HNE (PMAVVQSVP) conjugated to a pharmacokinetic modifier (PKM) and flanked with two fluorochromes VivoTag-S680 (Kossodo et al., 2011). This peptide, however, is also hydrolyzed by PR3 with similar potency. Despite this drawback, NE680 has been utilized to assess the activity of HNE in lung sections and for the *in vivo* imaging of HNE in mice challenged with N-formylmethionyl-leucyl-phenylalanine (fMLP) and lipopolysaccharide (LPS).

A year later, the same peptide sequence elongated at the N- and C-terminus with Q residues (QPMAVVQSVPQ) was incorporated in two other reagents for imaging HNE. In one of them, Nemo1, the peptide sequence was flanked with PEG (2), methoxycoumarin, and coumarin 343, while in the second one, Nemo2, the peptide sequence was flanked with PEG (2), TAMRA, and coumarin 343, and in addition, the peptide was lipidated with palmitic acid (Gehrig et al., 2012) (Table 3). Nemo2 was validated on RAW macrophages (which do not express HNE) supplemented with HNE, and the emission of fluorescence changed, reflecting the high FRET properties of donor-acceptor pairs and demonstrating the response to extracellular HNE. With this promising result, Nemo2 was applied in the measurement of primary mouse airway neutrophils from LPS-treated mice (Gehrig et al., 2012). The application of both probes led to the conclusion that during inflammation, HNE is predominantly active on the surface of infiltrated neutrophils. However, the QPMAVVQSVPQ peptide sequence is hydrolyzed by both NE and PR3, and therefore, we may consider that the detected fluorescence signal indicated both proteases.

**TABLE 3:** Structures of imaging reagents.

Structures of imaging reagents	References
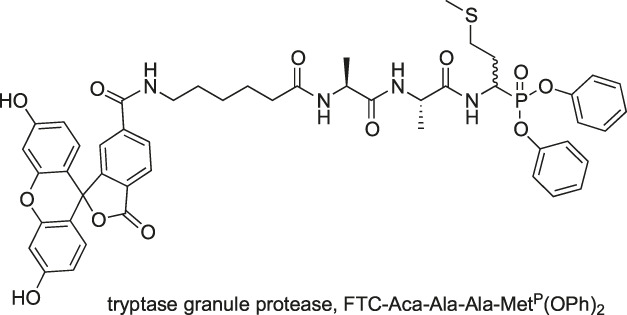	Abuelyaman et al. (1994)
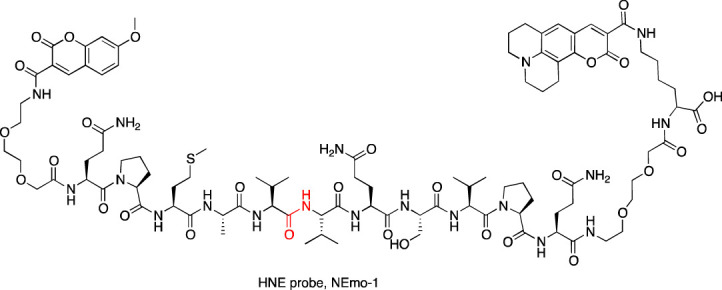	Gehrig et al. (2012)
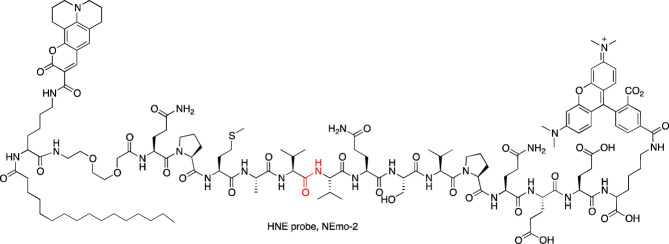	Gehrig et al. (2012)
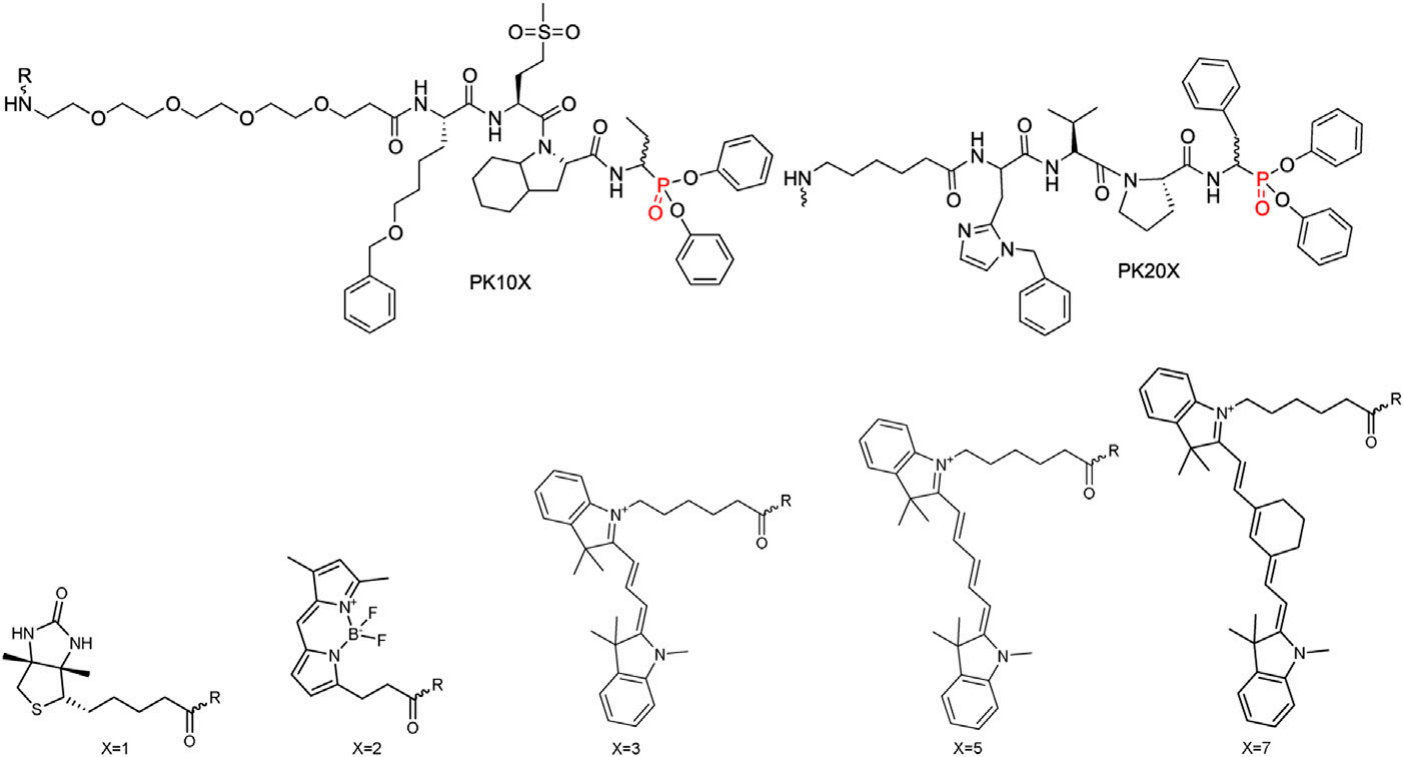	Kasperkiewicz et al. (2017), Kasperkiewicz et al. (2014)
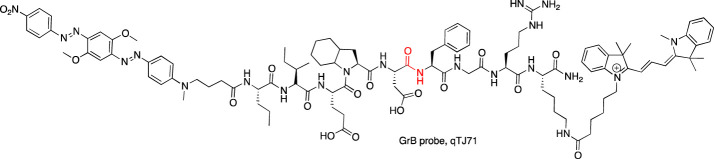	Janiszewski et al. (2020)
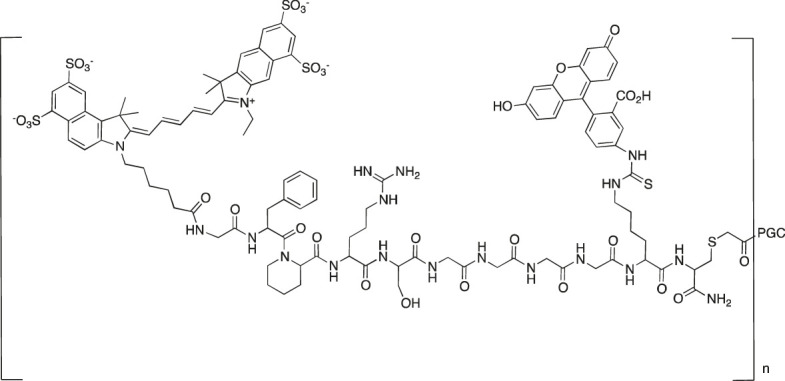	Jaffer et al. (2002), Tung et al. (2002)
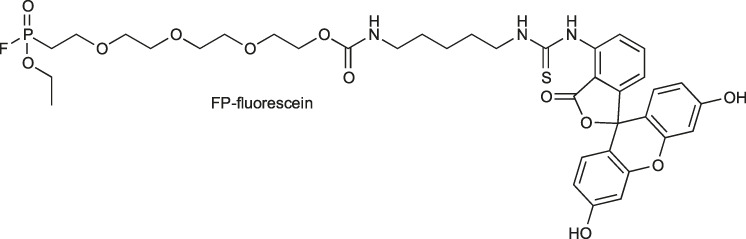	Liu et al. (1999)
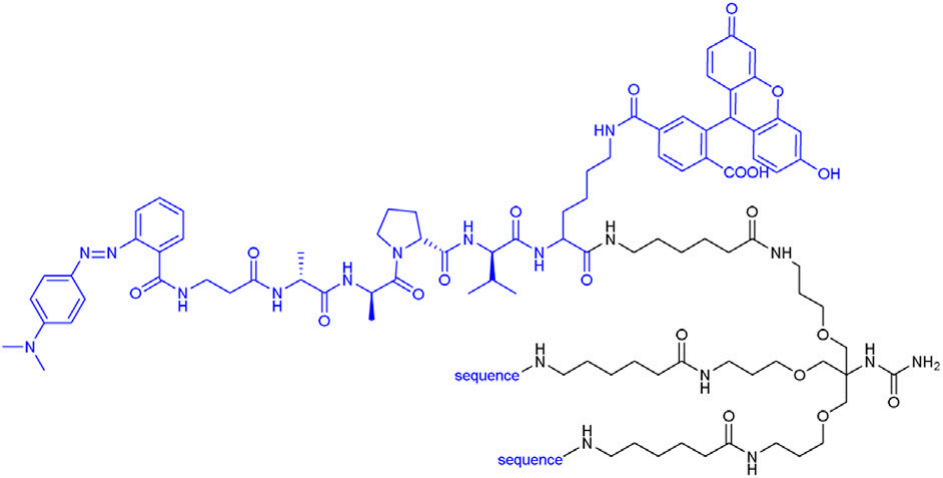	Craven et al. (2018)
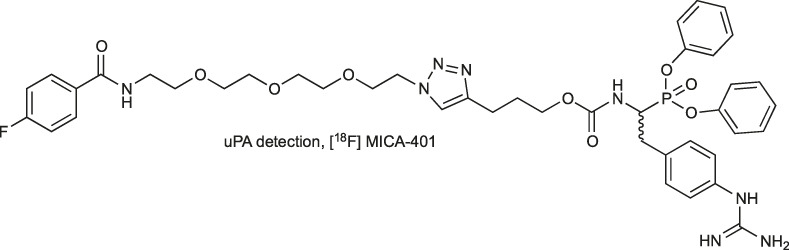	Ides et al. (2014)
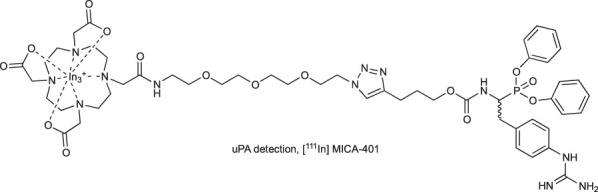	Vangestel et al. (2016)
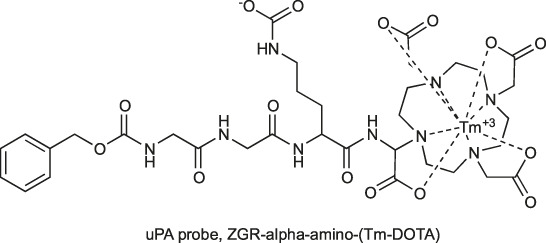	Yoo et al. (2014)
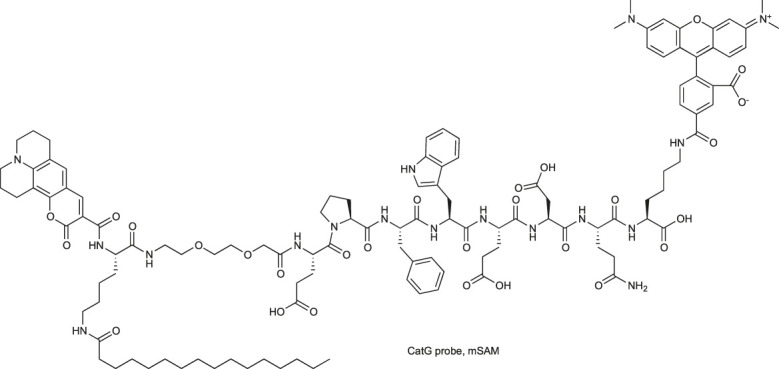	Guerra et al. (2019)

To circumvent the cross-reactivity of NE probes with PR3, the Drag group developed a new peptide sequence for HNE that incorporates only unnatural amino acid residues, guaranteeing probe selectivity. The first generation of chemical reagent with improved specificity to HNE was the biotinylated probe [biotin-PEG (4)-Nle (O-Bzl)-Met(O)_2_-Oic-Abu^P^(OPh)_2_, where Nle (O-Bzl) is 2-amino-6-benzyloxyhexanoic acid, Met(O)_2_ is l-methionine-sulfone, Oic is octahydroindole-2-carboxylic acid, Abu is 2-aminobutanoic acid, Table 3] that was applied in the imaging of HNE activity in neutrophil extracellular traps, one of the recently discovered techniques of neutrophil pathogen elimination, a type of cell death involving DNA release from the nucleus and the formation of traps to bind pathogens (Kasperkiewicz et al., 2014). Later, this group incorporated unnatural amino acids in ABPs for all NSPs and equipped them with four different fluorophores (BODIPY^FL^, Cy3, Cy5, and Cy7, Table 3), allowing the parallel imaging of four NSPs in neutrophils and demonstrating the uneven distribution of NSPs in neutrophil granules (Kasperkiewicz et al., 2017).

This study was extended, and the set of four probes was applied in the search for all NSP activity in NETosis. However, even fully processed NSPs were found to be catalytically inactive in DNA traps; therefore, their function in NETosis remains unclear (Kasperkiewicz et al., 2020). As applied ABPs are also inhibitors, they were used to study the role of NSPs in netosis, and by the inhibition of NSPs prior to NETosis induction it was demonstrated that NSPs are not crucial for this process and remain within NSP granules when induced with phorbol myristate acetate (PMA). The results obtained with biotinylated and fluorescent probes support the finding that there is a lack of active NSPs in neutrophil traps (Kasperkiewicz et al., 2020).

Additionally, another approach for HNE imaging in neutrophils was proposed by the Dhaliwal group. Here, an enzyme-activated reagent consists of three branches of the same substrate MR-Beta-Ala-AAPV-L (CF)-linker (connected to each other, Table 3). This tribranched scaffold guarantees fluorescence signal amplification only after probe hydrolysis (if excited); therefore, it was called a supersilent quencher sensor. Interestingly, when freshly isolated neutrophils were treated with the probe, no signal was observed, but in calcium ionophore-treated cells, an increase in fluorescence was detected. As this probe is substrate based, it allows the live imaging of enzyme activity changes in laser-scanning confocal microscopy. As previously described, a signal was detected only in stimulated cells (with *Pseudomonas aeruginosa*) (Craven et al., 2018).

In 2019, two CatG chemical markers were found to detect airway inflammation: first to monitor protease activity at the cell surface (mSAM) and second to measure enzyme activity in human body fluids (sSAM), such as blood, sputum supernatant and bronchial lavage. These substrate-based markers incorporate the EPFWEDQ sequence flanked by donor-acceptor pairs (TAMRA-coumarin 343) and differ only in the presence of lipid anchors (only in mSAM, Table 3). Evaluation of these markers demonstrate their utility in biochemical assays for CatG detection with flow cytometry and microscopy (Guerra et al., 2019).

Other serine proteases tightly packed in the cellular granules are Grs, which can be found in NK cells. Due to low substrate turnover and low enzyme concentration in cells, the chemical markers for these enzymes are rarely under investigation, even though one of the first imaged serine proteases was Grs in RNK-16 cells with a FTC-Aca-Ala-Ala-Met^P^(OPh)_2_ probe (Abuelyaman et al., 1994). As this peptide sequence possesses low specificity, Mahrus et al. changed the peptide sequence and developed two probes for detecting GrA and GrB (Mahrus and Craik, 2005). Although Grs have not been imaged, these tools provided one of the first valuable methods for Grs analysis. Later, Janiszewski et al. used unnatural amino acids to improve the potency of GrB phosphonate ABP and flanked a peptide sequence with Cy5. This probe allowed for GrB detection in cellular lysates of YT cells. As inhibitor-based probes can influence cellular function, a substrate-like reagent incorporating the same peptide sequence and Cy3-BHQ2 as a donor-acceptor pair was developed for GrB imaging. This reagent was successfully applied in granular GrB imaging in YT cells with high selectivity over caspases, which have overlapping specificity with GrB (Janiszewski et al., 2020).

An example of another serine protease whose activity can be imaged with a chemical marker is thrombin. Its activity has been detected in experimental thrombi with an NIR fluorescent probe that consists of a thrombin recognition peptide flanked with Cy5.5 and fluorescein isothiocyanate (FITC) (Cy5.5-Gly-D-Phe-Pip-Arg-Pro-Gly-Gly-Gly-Gly-Lys (FITC)-Cys-NH_2_) attached to the polymeric carrier (Jaffer et al., 2002). The construction of the probe allowed the *in vivo* imaging of thrombin activity within a trauma-induced hematoma or in an occlusive venous thrombus, where a fluorescence increase was observed after tail hematoma formation when compared with the control (Tung et al., 2002). As the probe was successfully used *in vivo*, it suggests its future application in elucidating the role of thrombin in thrombogenesis and other homeostatic and pathological conditions.

Another serine protease for which chemical markers are most commonly sought is uPA. One of the intriguing approaches for this enzyme investigation was proposed in 2013 by the Pagel group. In uPA substrate, the fluorophore moiety (AMC) was replaced with Tm-DOTA (Z-GGR-l-amino-(Tm-DOTA)), resulting in an enzyme-activated agent for uPA imaging. In this strategy, through probe hydrolysis by the target enzyme, a ^+^H_3_N-(Tm-DOTA) fragment is formed and can be monitored as a change in chemical exchange saturation transfer (CEST). As a control, the Eu-DOTA-Gly_4_, which is unresponsive to uPA activity, was used, and it was demonstrated that after injection of probes to the tail vein of the cancer bearing mouse, the CEST effect increased in tumor tissues right after injection to be later decreased more rapidly in Z-GGR-l-amino-(Tm-DOTA) then in control Eu-DOTA-Gly_4_ as a result of peptide cleavage by uPA. This was observed only locally in tumors, while in healthy tissues, the CEST effect did not change. With this, the authors demonstrated the utility of their chemical agent for adenocarcinoma detection (Yoo et al., 2014).

The other strategy to discriminate between high and low concentrations of uPA in tumors incorporates a radiolabeled probe [^111^In] MICA-401. The prototype probe consists of a phosphonate warhead with guanidine phenylalanine at P1 flanked by a PEG linker connected to the [^18^F]-4-fluorobenzoyl group (Ides et al., 2014). However, due to poor pharmacokinetics and cellular uptake, this reagent is not suitable for *in vivo* uPA imaging. Therefore, later, this molecule was modified, and the [^18^F]-4-fluorobenzoyl group was replaced with ^111^In radioisotope chelated by DOTA (Vangestel et al., 2016) (Table 3). Uptake of the indium complex was successfully detected with PET in two tumor models of cancer-bearing mice. However, high uptake in noninvaded lymph nodes was also observed, and it was concluded that this may represent a limitation to the clinical translation of this probe for oncological applications.

Specific probes are key factors for the adequate investigation of individual enzymes, but the generic probes possess broad reactivity toward all members of the serine protease family and are also essential tools for protease activity detection. For example, TAMRA-FP was used in the imaging of global serine protease activity in the tumor microenvironment of glioma brain cryosections (Aaltonen et al., 2020) (Table 3).

## Discussion and Perspectives

The majority of serine proteases, expressed as zymogens, require posttranslational processing to be active and play crucial roles in organisms. While inactive enzymes often play no functions, active enzymes become effectors and are involved in activation cascades important for not only physiological cellular function but also in the initiation and progression of some diseases.

The concentration of mature and active protease is therefore a better biomarker than the quantity of expressed enzyme. Detection, monitoring and imaging of active enzymes are essential for basic research and for personalized medicine. Therefore, recent studies in chemical biology, bioengineering, molecular biology and organic chemistry have focused on tools for active enzyme monitoring, namely, ABPs with broad applications in the study of individual proteases. The first attempts to find chemical tools for reliable indicators were made in 1994 by the Powers group, and ever since then, this technology has dramatically improved (Kam et al., 1993; Woodard et al., 1994). Recently, numerous attempts in peptidyl chemical markers for enzyme imaging were made, and despite their challenging development and some limitations, their broad application justifies the effort. One of the biggest challenges is to design chemical molecules that selectively label individual enzymes not only in a test tube but also in the cellular environment. Advances in peptide chemistry and the application of unnatural amino acids have spawned these reagents for more selective enzyme detection. In addition, inhibitor-like probes are equipped with a warhead, which in spite of irreversible binding, narrows the number of potential off target enzymes to one enzyme group; for example, phenylphosphonates label serine proteases, while acyloxymethylketones are selective toward cysteine proteases. This decreases nearly half of the enzymes with potential cross-reactivity. In substrate-based reagents, the specificity mainly relies on the peptide sequence, and all proteases recognizing the peptide sequence can potentially lead to undesired hydrolysis. Therefore, despite the many advantages of substrate-based probes, their development is more difficult compared with inhibitor-based probes, and for some proteases, current strategies might not be sufficient.

Overall, probes are designed to selectively address biological questions; hence, specificity guarantees specific labeling, but the biochemical selectivity does not always translate in cells and might depend on the context and target. This could be explained with an example. If a specific probe for the target protease possesses low potency and in addition this enzyme is characterized by a low turnover number, then in a complex system of cells, the hydrolysis product may be formed by other more abundant enzymes with broad substrate specificity and a high substrate turnover number (like metalloproteinases). In such a case, even minimal off-target hydrolysis of the probe results in false-positive output. Therefore, the selectivity should be taken in reference to the concentration tested. Additionally, the chemical marker potency is equally important to the selectivity, and for enzymes with a low turnover number, these features should be carefully analyzed. This makes the search for probes for low abundant enzymes with small substrate turnover the most challenging, and for these enzymes, new strategies would be beneficial.

Cell permeability is another limitation of the successful application of ABP. Some probes are cell membrane permeable for tested cells such as the PKX0X series (Kasperkiewicz et al., 2017), and they are probably delivered to the cell via pinocytosis. For some molecules, the lack of cell membrane permeability limits broad probe application. To increase the cell membrane penetration, probe molecules can be modified by the utility of a cell-penetrating peptide that is a short, positively charged peptide that can facilitate the cellular delivery of associated cargo along with probe. The probe cell-membrane permeability as well as its cytotoxicity is cell-specific, which on one hand is problematic since for every cell it has to be evaluated prior to application, but on the other hand, it can guarantee additional specificity to target cells.

To date, *in vivo* studies on inhibitor-based and substrate-based probes were performed on animal models, mainly mice. The translational success rate of animal studies to the human situation is not predictive and has to be carefully planned. For example, human and mouse GrA possess divergent catalytic preferences, and thus, probes designed for human GrA should not be applied in mouse models, while cathepsins possess a similar specificity across species, making the *in vivo* studies on mouse models with the chemical probe designed for human cathepsins more reasonable. Therefore, cathepsin probes have been successfully applied for tumor illumination in a mouse model; for example, probe 6QC was applied for *in vivo* surgical guidance and was detected intraoperatively with the da Vinci Si Surgical System (Bender et al., 2015; Yim et al., 2018). These results demonstrated large promise for the future treatment of tumors and their precise resection while saving healthy tissues. In addition, animal models may indirectly contribute to successful translation, providing information of mechanisms underlying the disease.

Activity-based probe profiling of serine proteases is less advanced than that of cysteine proteases, and therefore, there is still more to discover. However, our knowledge and efforts toward active cysteine protease profiling, especially cathepsins, give a strong basis for the hypothesis that similar applications can be made for serine proteases. Based on the recent developments, it seems that substrate-based probes or qABP for serine proteases are the future of active enzyme profiling within samples, as they were extensively used for the imaging of tumors (Weissleder, 2006; Withana et al., 2016; Withana et al., 2016), as previously mentioned for the 6QC probe (Bender et al., 2015). In tumor milieu, the elevated concentration of active enzymes is crucial for metastasis, tumor progression and angiogenesis. However, the higher infiltration of white blood cells, especially macrophages (TAMs) and neutrophils (TANs), was observed in the tumor microenvironment. Both cell TANs and TAMs were characterized with different phenotypes in tumor milieu, thus demonstrating their heterogeneity, and therefore, they could be applied as biomarkers for disease diagnosis.

TAMs have been under investigation for a long time, while to date, TANs seem to be neglected. This may be due to their sensitivity and easy activation during isolation, making the analysis of these cells challenging. However, neutrophils are the major cells containing serine proteases from the NSP group, and for now, their live imaging is not possible. Therefore, ABPs may facilitate the perfect solution for the imaging of TANs. Serine proteases are associated with many diseases, including neutropenia, cancer and lung diseases; therefore, the exact identification and measurement of their activity in pathological conditions is crucial for understanding their biology in these diseases. Imaging the activity of these proteases in patient samples will facilitate the diagnosis as well as evaluation of disease development, stage and response to treatment. Early diagnosis is a particularly important issue that allows rapid action toward treatment. Neutropenia is a disease that can be associated with mutations in one of the serine protease genes, ELANE. However, there are many types of neutropenia, and depending on the mutation, a different treatment strategy should be applied. To date, neutropenia diagnosis is based on the clinical manifestations, circulating neutrophil count and, less often, bone marrow, genetic and immunological analyses that are time consuming and require many blood samples. Therefore, new methods for treating and diagnosing neutropenia are the object of extensive research, and ABPs for NSPs may be a holy grail for diagnosing this disease. To date, one of the serine proteases is already successfully used in prostate cancer diagnosis. KLK-3 is a screening biomarker and an indicator of disease progression, and it has been studied extensively, while the other enzyme from this family, KLK-6, has been analyzed as a biomarker for ovarian cancer. In fact, the diagnosis of prostate cancer in blood tests has dramatically improved its detection, and other enzymes from this family are currently under investigation as potential markers for diagnosing cancer.

Most studies on imaging tools have focused on cysteine proteases, while serine proteases seem to be neglected, and there is still much to be discovered. To date, the vast majority of reagents dedicated to serine proteases have been developed to study the activity of NSPs, uPA and KLKs; however, the activities of only a few enzymes from this group have been visualized. The application of chemical reagents for enzyme activity studies allow the analysis of the activity of several enzymes in the same cell or organism in parallel. In this review, available peptide-based techniques for serine protease imaging are discussed, including the critical view of their design. Currently, there are two main types of chemical markers for serine protease imaging, and they are based on the substrate or inhibitor structure. Classic ABPs are built from three main functional elements: warheads, peptide sequences and tags, while in substrate-based reagents, peptide sequences are flanked with a fluorescence donor-acceptor pair. In both strategies, for labeling and consequent imaging, enzyme activity is required. Above all, this is the main advantage of chemical markers over antibody detection. This feature can be applied in the study of enzyme functionality, activation, localization and inactivation on a cellular level but also in the whole organism for tumor detection. Proteases often play a key role in tumors, and their accurate detection may serve as a diagnostic method, while their illumination in the body can help in the precise removal of diseased tissue.

## Concluding Remarks

• activity-based reagents can specifically detect individual active proteases.
• enzyme-activated markers are more adequate chemical tools for live imaging than inhibitor-based probes.
• activity-based reagents might be applied as diagnostic and prognostic markers.
• activity-based reagents can be used to monitor tumor milieu.
• activity-based reagents can be used to monitor tumor-associated white blood cells, namely, macrophages and neutrophils.
